# Association Studies and Legume Synteny Reveal Haplotypes Determining Seed Size in *Vigna unguiculata*

**DOI:** 10.3389/fpls.2013.00095

**Published:** 2013-04-15

**Authors:** Mitchell R. Lucas, Bao-Lam Huynh, Patricia da Silva Vinholes, Ndiaga Cisse, Issa Drabo, Jeffrey D. Ehlers, Philip A. Roberts, Timothy J. Close

**Affiliations:** ^1^Department of Botany and Plant Sciences, University of California RiversideRiverside, CA, USA; ^2^Department of Nematology, University of California RiversideRiverside, CA, USA; ^3^Senegalese Institute of Agricultural ResearchThiès, Senegal; ^4^Institute of Environmental and Agricultural ResearchOuagadougou, Burkina Faso

**Keywords:** genome-wide association study, QTL analysis, single nucleotide polymorphism, cowpea, seed size, comparative genomics

## Abstract

Highly specific seed market classes for cowpea and other grain legumes exist because grain is most commonly cooked and consumed whole. Size, shape, color, and texture are critical features of these market classes and breeders target development of cultivars for market acceptance. Resistance to biotic and abiotic stresses that are absent from elite breeding material are often introgressed through crosses to landraces or wild relatives. When crosses are made between parents with different grain quality characteristics, recovery of progeny with acceptable or enhanced grain quality is problematic. Thus genetic markers for grain quality traits can help in pyramiding genes needed for specific market classes. Allelic variation dictating the inheritance of seed size can be tagged and used to assist the selection of large seeded lines. In this work we applied 1,536-plex SNP genotyping and knowledge of legume synteny to characterize regions of the cowpea genome associated with seed size. These marker-trait associations will enable breeders to use marker-based selection approaches to increase the frequency of progeny with large seed. For 804 individuals derived from eight bi-parental populations, QTL analysis was used to identify markers linked to 10 trait determinants. In addition, the population structure of 171 samples from the USDA core collection was identified and incorporated into a genome-wide association study which supported more than half of the trait-associated regions important in the bi-parental populations. Seven of the total 10 QTLs were supported based on synteny to seed size associated regions identified in the related legume soybean. In addition to delivering markers linked to major trait determinants in the context of modern breeding, we provide an analysis of the diversity of the USDA core collection of cowpea to identify genepools, migrants, admixture, and duplicates.

## Introduction

**Figure 1:**
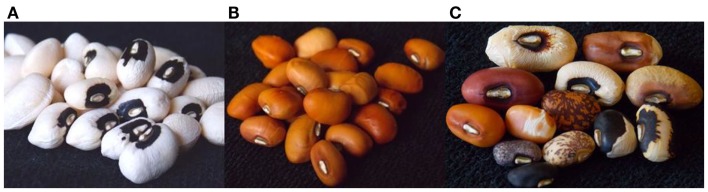
**Popular cowpea seed types include “blackeyed” and “buff” represented by (A) California Blackeye 27 and (B) IT82E-18**. However, a diversity of cowpea seed types exist **(C)**.

Cowpea is a warm-season legume grown throughout the tropics and several areas of the subtropics. West African countries led by Nigeria and Niger produce 70% of the world’s crop on 10 million ha (FAOSTAT, 2013). The North-Eastern region of Brazil is the second largest region of production, followed by Eastern and Southern Africa, South Asia, and North America. As is the case for other grain legumes, farmers’ and market acceptance of cowpea are driven by the visual appearance of the grain. In most markets, large seed size is desirable and this is reflected in price premiums for large cowpea grain. Across Africa a diversity of grain sizes and colors exist which have varying importance in local or regional contexts. In West Africa the two most important grain types are large white or brown with rough seed coat texture, while in East and Southern regions of Africa relatively smaller seeds with smooth texture and brown to red color predominate in markets. In the Western United States, Southern Europe, and the Middle-East the “blackeyed pea” cowpea predominates. This type of cowpea is characterized by a large grain and white seed coat with a pigmented “eye” around the hilum. Figure 1 displays a diversity of cowpea seed types. “Fresh-shell” varieties are also desired which are harvested before maturity for their large seed that can be easily removed from green pods. Consumer preference primarily demands large seed when grown for grain; however, small seed is preferred when seed is sold by volume for use as a fodder or cover crop.

Seed size has several agronomically important impacts. Large seeded cowpea have enhanced emergence when planted deep (up to 5 cm), tend to emerge earlier, and produce larger plants during early development (Lush and Wien, 1980). In contrast, while large seeds typically have advantages over small seeded competitors (Wulff, 1986), small seeds are desirable for early drought conditions because they are able to transpire less water relative to their ability to reach water supplies (Hendrix et al., 1992). This may be particularly important for semi-arid rainfed growing regions.

Seed size is a very stable component of grain yield with high heritability for many crop plants including wheat (Giura and Saulescu, 1996), soybean (Cober et al., 1997), cowpea (Drabo et al., 1984), and mung bean (Fery, 1980). Several genes are known to impact the inheritance of seed size in cowpea. Drabo et al. (1984) proposed that at least eight loci contribute to the quantitative inheritance of seed size and Fatokun et al. (1992) identified two major, unlinked genomic regions, one of which is orthologous to a seed size QTL in mung bean. The orthology of this locus was later confirmed by its identification and association to seed size in soybean (Maughan et al., 1996). Exploration of legume synteny for cowpea trait characterization continues to be a rewarding approach that has also been used to better describe resistance to fungal pathogens (Muchero et al., 2011; Pottorff et al., 2012a), tolerance to heat during reproductive development (Lucas et al., 2013a), and leaf morphology (Pottorff et al., 2012b).

The introgression of novel traits from diverse collections typically compromises seed size among progeny. Because of the importance of grain size in market appeal, recovery of adequate grain size is an important objective following elite × exotic crosses. Wide crosses are commonly pursued to help deliver new varieties with enhanced resistance to biotic and abiotic stress. Several cycles of backcrossing help recover elite characteristics including seed size; however, this process can be cumbersome and inefficient due to possible linkage drag and the polygenic nature of the trait. To help improve the selection of desirable lines we developed associations between genic SNP markers and seed size using experimental populations, a diversity collection, and knowledge of legume synteny.

## Materials and Methods

### Phenotype data

Seed size was calculated as weight per 100 seed. The seeds we measured were harvested from plants grown under favorable conditions whether in the field or in the greenhouse. This means that plants were well watered and treated with pesticides as needed. The populations which were used are presented in Table 1 and are among those used to develop the consensus genetic map of cowpea (Lucas et al., 2011). All populations were at least at the F_8_, except the IT84S-2246 × Mouride population which was phenotyped and genotyped at the F_4_ generation. All eight populations were grown in the greenhouse, while the CB27 × IT82E-18 and CB46 × IT93K-503 populations were also grown in field trials. The CB27 × IT82E-18 population was grown during the summers of 2010 and 2011 at the University of California Riverside Citrus Experiment Station in Riverside, Riverside, CA, USA. The CB46 × IT93K-503 population was grown during the summer of 2008 at two field stations led by (1) the Senegalese Institute of Agricultural Research (ISRA/CNRA) in Bambey, Senegal and (2) the Institut de l’Environnement et de Recherches Agricoles (INERA) at Kamboinse, Burkina Faso. In field trials ∼100 seeds per 6-m plot were planted in four replicates for each sample. In all greenhouse and field trials mature pods were harvested and dried for storage (<15% moisture). Seeds were subsequently cleaned from the pods, counted, and weighed to determine the weight of a random sample of 100 seeds. Seed size data provided online by Germplasm Resources Information Network (USDA-ARS and National Genetic Resources Program, 2013) was used for genome-wide association mapping.

**Table 1:** **Characteristics of eight bi-parental populations of cowpea used to associate loci with seed size**.

Parents		Population size*	Number of polymorphic SNPs^†^	Population 100 seed weight (g)	
				Range	Average
CB27	IT82E-18	160	430	8.53–30.96	17.58
CB27	UCR 779	56	560	9.57–29.81	18.28
CB27	24-125-B-1	87	329	9.98–28.50	17.54
CB46	IT93K-503	114	374	7.99–34.95	17.00
Dan Ila	TVu-7778	79	288	3.26–19.50	12.77
524B	IT84S-2049	85	438	12.55–24.15	17.29
TVu-14676	IT84S-2246	136	345	5.61–30.51	15.59
IT84S-2246	Mouride	87	347	12.55–22.35	16.70

*Number of samples used for QTL analysis after eliminating rogues.

*^†^Number of polymorphic SNPs out of 1,536 genotyped SNPs which could be mapped*.

### Genotype data

The 1,536-plex EST-derived SNP genotype data used to build the consensus genetic map of cowpea (Lucas et al., 2011) was also used to perform QTL analyses of the eight bi-parental populations. Genotype data for 171 individuals of the USDA core collection of cowpea (USDA Core) (Gillaspie et al., 1996) were also obtained using the 1,536-plex genotyping platform developed by our group (Muchero et al., 2009). SNP calls were exported for further processing from the Illumina GenomeStudio software (Illumina, 2010). Rogue individuals among the bi-parental populations which were described in Lucas et al. (2013b) were removed prior to QTL analysis. Similarly, genotype data for the USDA Core were used to identify and remove duplicate individuals using ParentChecker (Hu et al., 2012). ParentChecker was also helpful for formatting files for downstream analyses. SNPs were filtered on the basis of minor allele frequency (>0.20 for QTL and >0.10 for GWAS and analysis of population structure) to develop a set of polymorphic markers appropriate for analyses. The genotype data for the USDA core are provided in the Section “USDA Core Genotypes” in Supplementary Material.

### Marker-trait associations

QTL IciMapping (Li et al., 2008) was used to perform inclusive composite interval mapping for seed size based on 100 seed weight data from eight bi-parental mapping populations. In a method similar to Lucas et al. (2013a), the genetic map used for QTL analyses was a composition of population specific map marker orders and distances, and consensus linkage groups assignments. Regions of the genome contributing major QTL were identified after considering (1) regions with LOD scores >3.0; (2) effect size >15% of phenotypic variance explained; (3) marker density; (4) span of the trait-associated region; (5) discovery in multiple populations or *via* GWAS; (6) haplotype consistency when QTL were discovered in multiple populations; and (7) homology with trait-associated regions in soybean. The potential effect of stacking favorable alleles for multiple QTL was also investigated by grouping lines based on their QTL composition. This was done for populations in which multiple QTLs were discovered. Individuals with no, one, or several favorable alleles underlying the seed size QTLs we report were grouped and the average seed size was determined for that group and compared to the population average. A single factor analysis of variance was performed to determine if differences in seed size were due to QTL content. The ICIM-EPI function within QTL IciMapping (Li et al., 2008) was used to search for QTL interactions.

Six-hundred and sixty-five EST-derived SNP markers with minor allele frequency >0.10 that were located among unique bins (one marker per bin) of the cowpea consensus genetic map were used to identify population structure of the subset of the USDA core. STRUCTURE (Pritchard et al., 2000) was used with BURNIN = 10,000 and NUMREPS = 50,000, with five runs of *K* = 1–15. The Evanno method (Evanno et al., 2005) facilitated by STRUCTURE HARVESTER (Earl and vonHoldt, 2012) was used in addition to CLUMPP (Jakobsson and Rosenberg, 2007) and DISTRUCT (Rosenberg, 2004) to reconcile genepools on the basis of geographic collection information provided online by GRIN (USDA-ARS and National Genetic Resources Program, 2013). Whole genome ancestry estimates (*Q*-matrix) computed from multiple STRUCTURE runs by CLUMPP were used as a covariate in the generalized linear model of association mapping provided by TASSEL 3.0 (Bradbury et al., 2007). Markers that showed −Log(*P*-Values) >3.0 that were also identified using the bi-parental populations were considered significantly associated.

### Synteny analysis

Regions of the soybean genome syntenic with the cowpea seed size QTLs reported here were searched for seed size QTLs. HarvEST: cowpea (Wanamaker and Close, 2011) was used to identify synteny based on BLASTX scores (<10^−10^) between cowpea unigenes containing mapped SNPs and translated gene models from soybean (Schmutz et al., 2010). Soybean genomic locations homeologous to cowpea seed size QTL were reconciled with an abundance of soybean seed size QTL inventoried and integrated with the physical genome by SoyBase (Grant et al., 2010). Only soybean QTL that were within or tightly linked to the syntenic region (<3 million base pairs) were considered orthologous.

## Results

### Field and greenhouse trials

**Figure 2:**
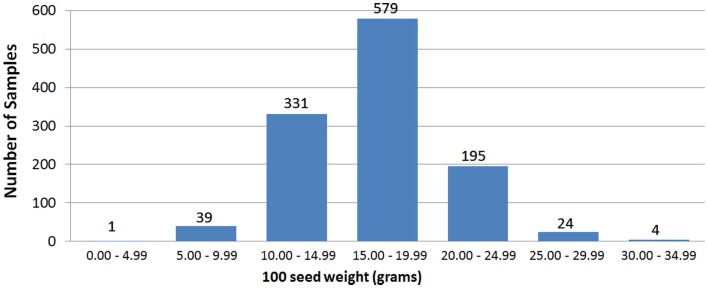
**Phenotypic distribution of seed size among eight bi-parental populations of cowpea**.

The two field trials using the CB27 × IT82E-18 population produced seed size data that were strongly correlated to each other (Pearson’s *r* = 0.83) and similar to that of the greenhouse trial (*r* = 0.59 and 0.63 for 2010 and 2011 respectively). This was also the case for the multiple trials of the CB46 × IT93K-503 population where field trials were correlated to each other (*r* = 0.30) and more so to the greenhouse trial (*r* = 0.52 and 0.31 for ISRA and INERA trials respectively). Figure 2 provides the phenotypic distribution of seed size among all eight bi-parental populations. The smallest seeded line had a 100 seed weight of 3.26 g and was produced by the parents Dan Ila and TVu-7778. The largest seeded line was produced by CB46 and IT93K-503 and had a 100 seed weight of 34.06 g. The average seed of an individual from the eight bi-parental populations had a 100 seed weight of 15.50 g. Phenotypic distributions of seed size for each trial are provided in Section “Population Seed Sizes” in Supplementary Material. Seed sizes for the parents of the mapping populations are provided in Section “Parent Seed Sizes” in Supplementary Material which ranged from 11.60 to 26.41 g per 100 seed. Phenotypic and genotypic characteristics of the mapping populations are provided in Table 1.

### Association studies

Ten QTL for seed size, representing ∼10% of the mapped cowpea genome were identified among the eight bi-parental populations (Table 2). Most had narrow spans (<5 cM), accounted for a substantial proportion of the phenotypic variance (average of 30%), and were associated with multiple SNP markers (average LOD > 8.5). LOD score traces for each QTL discovery are included in the Supplementary Material (“RIL QTL LOD”). Haplotypes associated with large and small seed were consistent among discovery populations when QTL were detected in multiple populations (“Alleles” in Supplementary Material). This is the situation for *Css* – *1* where markers 1_0974 and 1_0078 were detected among different experiments. Allelic variation important for seed size can be found among all parents of the bi-parental populations except for Dan Ila and IT84S-2049. The additive allelic effect of *Css* – *1* was similar (1.77 and 2.18 g) between multiple trials of the CB27 × IT82E-18 population. This is also true for the multi-trial detection of *Css* – *2* using the CB46 × IT93K-503 population (1.97 g for both experiments).

**Table 2:** **Ten seed size QTL identified among eight bi-parental populations of cowpea**.

QTL name	Discovery population(s)	Large seed allele donor(s)	LOD	%Phe	Additive (g)	Linkage group	QTL location (cM)	Number of markers
Css – 1*	CB27 × IT82E-18	IT82E-18	3.82–34.43	24.6–45.0	1.77–3.34	5	53.23–57.30	5
	CB27 × UCR 779	UCR 779	
	TVu-14676 × IT84S-2246	TVu-14676	
Css – 2^†^^§^	CB27 × IT82E-18	CB27	4.64–7.94	4.2–17.2	1.03–1.97	7	18.70–22.68	6
	CB46 × IT93K-503	CB46	
	TVu-14676 × IT84S-2246	TVu-14676	
Css – 3^†^	524B × IT84S-2049	524B	2.94–6.63	5.2–25.2	0.69–1.30	2	18.92–32.75	6
	IT84S-2246 × Mouride	IT84S-2246	
	TVu-14676 × IT84S-2246	TVu-14676	
Css – 4^†^^§^	CB27 × IT82E-18	CB27	5.37–5.42	9.9–26.8	1.12–1.16	6	31.28–57.41	4
	IT84S-2246 × Mouride	Mouride	
Css – 5^‡^^§^	CB46 × IT93K-503	IT93K-503	5.30	41.1	1.70	8	55.20–61.60	2
Css – 6^†^^§^	Dan Ila × TVu-7778	TVu-7778	3.38–3.69	20.3–40.8	1.24–1.38	10	0.00–4.90	3
	IT84S-2246 × Mouride	Mouride	
Css – 7^†^^§^	CB46 × IT93K-503	CB46	4.07	15.0	1.95	2	56.95–62.06	2
Css – 8^†^^§^	CB27 × 24-125B-1	24-125B-1	5.18	26.7	1.80	6	3.82–4.55	2
Css – 9^†^	CB27 × 24-125B-1	24-125B-1	3.24	16.6	1.42	5	18.50–21.57	2
Css – 10^‡^^§^	CB46 × IT93K-503	CB46	10.4	46.7	1.66	7	31.40–32.20	2

*Statistical tests used to identify QTL from the discovery experiments are reported for the Log of Odds score (LOD), percent of phenotypic variation explained by the QTL (%Phe), and the absolute value of the additive effects (Additive). Linkage group and centi-Morgan (cM) positions were identified from the consensus genetic map of cowpea. The number of markers used to tag a QTL among the discovery experiments is also included. Underlined QTL were also identified in the genome-wide association study*.

*Indicates the QTL was discovered by analyzing data from both field and greenhouse experiments.

*^†^Indicates the QTL was discovered by analyzing data from greenhouse experiments*.

*^‡^Indicates the QTL was discovered by analyzing data from field experiments*.

*^§^ Indicates the QTL is supported based on synteny to soybean seed size associated loci*.

“Multi-QTL Effect” in Supplementary Material displays the potential for genetic gain by combining favorable alleles for multiple QTLs. QTL content has the most significant effect on seed size for the CB27 × IT82E-18 population *F*(3, 149) = 28.51, *p* = 1.25E-14, η^2^ = 0.36). This is also true for all other populations except for the CB46 × IT93K-503 population where groups based on QTL content are mainly different due to chance *F*(4, 86) = 1.29, *p* = 0.28, η^2^ = 0.06. See Section “Multi-QTL Effect” in Supplementary Material for test statistics for all populations. No significant QTL interactions were found among the discovery experiments.

**Figure 3:**
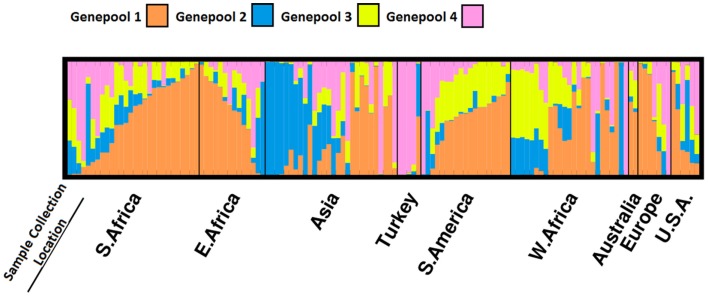
**Population structure underlying a subset of the USDA core collection of cowpea**. Samples are first sorted based on their geographic location of collection and then sorted based on a coancestry matrix with *K* = 4.

Six-hundred and sixty-five SNPs which were polymorphic among 171 accessions of the USDA core were used to identify 27 duplicated accessions (“USDA Core Duplicates” in Supplementary Material). An additional 10 accessions were excluded from further analysis due to a lack of geographic collection information. This filter yielded 134 accessions appropriate for population structure and association analyses. Geographic collection information and the Evanno method (“Evanno Method” in Supplementary Material) supported four subpopulations which accounted for a substantial proportion of population structure underlying the USDA core (Figure 3). Genepool 1 was the most prolific and was comprised of a majority of the samples collected in Eastern and Southern Africa. Samples collected in Asia were categorized primarily in genepool 2, and West Africa and Turkey were identified as genepool 3 and genepool 4, respectively. The genomes of 46 samples were primarily derived from genepool 1 and up to 87 samples contained a substantial proportion originating from genepool 1 (“Merged *Q*-Matrix” in Supplementary Material). While only 8 samples could be attributed entirely to genepool 3, 43 samples were admixed with genepool 3. Samples collected in South America were almost always an admixture of genepools 1 and 3. Most of the migrants were collected in West Africa and Asia.

**Figure 4:**
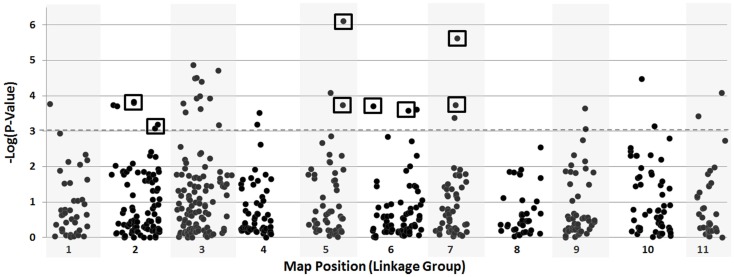
**Genome-wide association analysis of seed size using the USDA core collection of cowpea**. Loci surpassing significance thresholds that were also associated with seed size among the bi-parental populations are boxed.

Thirty-six SNP loci used in the GWAS of the USDA core surpassed −Log(*P*-value) thresholds and confirmed six of the ten QTL proposed by the bi-parental populations (Figure 4). This information is incorporated into Table 2 and is more comprehensively provided in Section “GLM” in Supplementary Material.

### Synteny

Based on the syntenic relationships described by Lucas et al. (2011), 7 out of the 10 QTL identified in the bi-parental populations were supported by knowledge of seed size in soybean (Tables 2 and 3). A total of 19 associations between markers and seed size developed in soybean (Orf et al., 1999; Csanadi et al., 2001; Specht et al., 2001; Hoeck et al., 2003; Hyten et al., 2004; Zhang et al., 2004; Panthee et al., 2005; Reinprecht et al., 2006; Chen et al., 2007; Gai et al., 2007) were in regions homoeologs to the cowpea seed size QTL reported here. More details of the synteny analysis are presented in Section “Synteny Analysis” in Supplementary Material.

**Table 3:** **Seven QTL controlling the inheritance of seed size in cowpea are syntenic to regions with known association to seed size in soybean**.

Cowpea	Soybean			Soybean seed size associations		
QTL	Chr	Start	End	QTL1	QTL2	QTL3
Css–2	1	1484033	3411009	Sdwt 18-1.2	–	–
	9	39678719	40435370	Sdwt 10-10	Sdwt 15-6	–
Css–4	19	48637729	50058893	Sdwt 12-3	Sdwt 13-9	–
	9	39678719	40435370	Sdwt 10-10	Sdwt 15-6	–
Css–5	4	1776391	1787115	Sdwt 13-4	–	–
Css–6	7	185839	2305626	Sdwt 10-11	Sdwt 7-6	–
	8	15355701	17919999	Sdwt 22-1	–	–
Css–7	10	45955504	47689454	Sdwt 25-4	–	–
	20	35530502	38505007	Sdwt 15-5	Sdwt 24-3	–
Css–8	8	14338579	15148040	Sdwt 22-1	–	–
Css–10	11	5421236	5680908	Sdwt 21-1	Sdwt 22-2	Sdwt 25-2
	1	49378273	49834904	Sdwt 15-2	Sdwt 18-1.1	Sdwt 7-4

*Chromosome (Chr) and base pair starting (Start) and ending (End) positions in the soybean genome are indicated for each syntenic relationship. Up to three soybean QTL can be found within or tightly linked (<3 Mbp) to the syntenic span; except Sdwt 18-1.1, 21-1, 22-2, and 25-2 which are <5 Mbp*.

## Discussion

**Figure 5:**
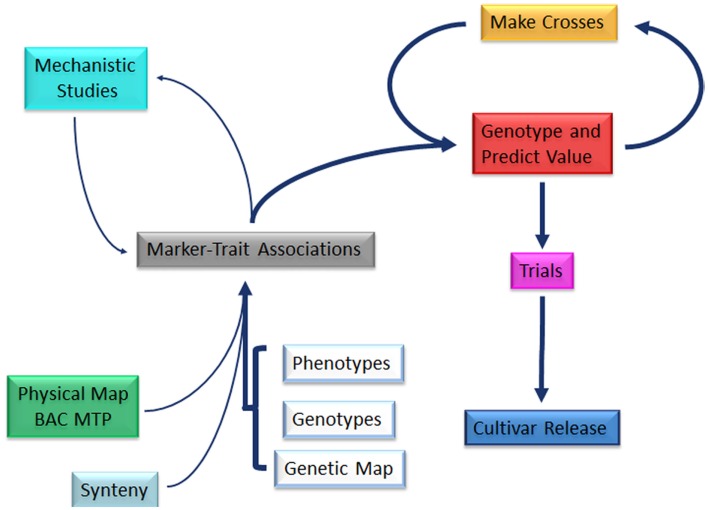
**General pathway of marker-assisted breeding strategies which rely heavily on the development of marker-trait associations**.

Cowpea with specific seed size can be predicted on the basis of marker-trait associations. These associations provide a foundation for marker-assisted breeding and can be developed through QTL analysis and association mapping which couple phenotypes, genotypes, and a genetic map (Figure 5). DNA markers tagging allelic variation underlying seed size QTL can be used to track trait determinants among breeding cycles. This approach facilitates the simultaneous improvement of a variety for different traits of interest.

From the standpoint of breeding, the most applicable association studies assess broad pedigrees and tag associated genomic regions with dense markers. Marker-trait associations identified in one population may not segregate or contribute to the inheritance of the trait in a different population. To support new marker-trait associations we used multiple populations, two popular methodologies (QTL and GWAS), and knowledge of seed size in soybean. The intent of this study was to assess allelic variation important for the inheritance of seed size in cowpea primarily in the context of marker-assisted breeding and comparative genomics. The associations developed in this study would be best validated after years of using them in breeding; however, we feel this work provides an important framework for future breeding initiatives and explores the potential of genomics to help deliver new varieties of cowpea. The accuracy of these marker-trait associations could be assessed by comparing the estimated additive allelic effects reported here with realized gains after using these markers for selection.

Based on our analyses there is a large potential to produce larger-seeded lines by combining favorable alleles for multiple QTLs. However, our analysis of this potential is limited because our study lacked recombinants for all possible QTL combinations. A more complete view could be provided by studying the behavior of QTLs outside of their discovery pedigree. This could be accomplished by pursuing a mating scheme which used lines from different pedigrees and with different QTL content.

Breeders interested in using marker-trait associations would benefit from knowledge of linkage between trait determinants. The locations of the QTLs reported here are mainly unlinked or distantly linked to other traits characterized using the consensus genetic map of cowpea, including heat tolerance during reproductive development (Lucas et al., 2013a), leaf morphology (Pottorff et al., 2012b), and resistance to *Fusarium oxysporum* f.sp. *tracheiphilum* race 3 (Pottorff et al., 2012a). One (*Thr* – *1*) of the three QTL known to impact resistance to feeding damage caused by foliar thrips (*Thrips* *tabaci* and *Frankliniella schultzei*) (Lucas et al., 2012) overlaps with a seed size QTL (*Css* – *3*) reported in the current work. The markers within this overlapping region include 1_0164 and 1_0589 where genotypes homozygous for AA at these were associated with large seed and thrips resistance. This means it would require a rare recombination to break the linkage between resistance to foliar thrips conferred by *Thr* – *1* and a small seed conferred by *Css* – *3*. Other overlaps can be found between seed size QTL and regions associated with *Macrophomina phaseolina* resistance (Muchero et al., 2011) (*Css* – *9* with *Mac* – *6*, and *Css* – *4* with *Mac* – *8*). Therefore using markers linked to these overlapping regions may simultaneously affect seed size and resistance to *Macrophomina*. In such regions a higher density of markers would be useful for marker-assisted breeding.

SoyBase (Grant et al., 2010) is an excellent resource for legume researchers. The integration of QTL studies with the physical map made it possible for us to survey commonalities among association studies performed in plants of different genera. Such knowledge may provide paths for mechanistic studies aiming to pinpoint trait determinants. From the standpoint of this study, the co-localization of seed size QTL in soybean and cowpea provides a level of validation for new marker-trait associations. Knowledge of legume synteny and trait determinants would be enhanced by developing resources similar in density to the soybean community for other legumes (i.e., common bean, cowpea, mung bean, peanut, chickpea, etc.). An agricultural project that would be coordinated among groups with expertise in different legumes could greatly enhance comparative resources and the efficiency of new initiatives.

The fact that our study uses approaches capable of clarifying the domestication history and dispersal of modern cowpea does not escape our attention; however, due to sample size we advocate a conservative interpretation of the diversity analysis using the USDA core as presented here. Rather than focusing on potential insight concerning cowpea domestication or proposing new marker-trait associations, we present the results of the genome-wide association study only to provide a modest assessment of collection diversity and to help support QTL identified among the bi-parental populations. The International Institute of Tropical Agriculture maintains a diverse collection which has been previously characterized on the basis of geographic, agronomic, and botanical descriptors (Mahalakshmi et al., 2007), but no collection of cowpea has been viewed in light of dense genotype data. The financial costs required for genotyping the rest of the USDA core is inexpensive relative to the value of the insight that can be gained. From our analysis of a small subset (171 samples) of the entire USDA core (720 samples) we were able to identify many duplicated accessions (∼17%), overrepresentation of the South/East African genepool, and we were able to perform an association study which mainly agreed with the QTL studies stemming from the bi-parental recombinant-inbred populations. SNP data from the entire core collection could be used to improve the diversity collection and its impact on the cowpea community. Phenotypic data for a number of traits are available on GRIN and could be combined with genotype data, similar to this work, to facilitate the discovery of numerous marker-trait associations. The use of historical data would be a cost effective approach to improve knowledge of cowpea genetic diversity and allelic variation contributing to the inheritance of agronomically important traits. The feasibility of this approach was recently supported within the barley community (Wang et al., 2011). That work helped demonstrate the utility of historical data after careful consideration of population size and experimental design. Furthermore, a comparative analysis of the diversity among core collections (i.e., IITA, USDA, and UCR) would be valuable for identifying instances of ascertainment bias and duplicated accessions possibly known by different names. This is a documented issue for U.S. collections (Vigna Crop Germplasm Committee, 1996), and continued application of genotype data to identify duplicates would be particularly helpful in cutting costs associated with the maintenance of collections and for designing new experiments.

## Conflict of Interest Statement

The authors declare that the research was conducted in the absence of any commercial or financial relationships that could be construed as a potential conflict of interest.

## Supplementary Material

The Supplementary Material for this article can be found online at: http://www.frontiersin.org/Plant_Genetics_and_Genomics/10.3389/fpls.2013.00095/abstract
